# Propionic Acidemia, Methylmalonic Acidemia, and Cobalamin C Deficiency: Comparison of Untargeted Metabolomic Profiles

**DOI:** 10.3390/metabo14080428

**Published:** 2024-08-02

**Authors:** Anna Sidorina, Giulio Catesini, Elisa Sacchetti, Cristiano Rizzo, Carlo Dionisi-Vici

**Affiliations:** Division of Metabolic Diseases and Hepatology, Bambino Gesù Children’s Hospital IRCCS, 00146 Rome, Italy; giulio.catesini@opbg.net (G.C.); elisa.sacchetti@opbg.net (E.S.); cristiano.rizzo@opbg.net (C.R.); carlo.dionisivici@opbg.net (C.D.-V.)

**Keywords:** methylmalonic acidemia, propionic acidemia, cblC deficiency, untargeted metabolomics

## Abstract

Methylmalonic acidemia (MMA), propionic acidemia (PA), and cobalamin C deficiency (cblC) share a defect in propionic acid metabolism. In addition, cblC is also involved in the process of homocysteine remethylation. These three diseases produce various phenotypes and complex downstream metabolic effects. In this study, we used an untargeted metabolomics approach to investigate the biochemical differences and the possible connections among the pathophysiology of each disease. The significantly changed metabolites in the untargeted urine metabolomic profiles of 21 patients (seven MMA, seven PA, seven cblC) were identified through statistical analysis (*p* < 0.05; log2FC > |1|) and then used for annotation. Annotated features were associated with different metabolic pathways potentially involved in the disease’s development. Comparative statistics showed markedly different metabolomic profiles between MMA, PA, and cblC, highlighting the characteristic species for each disease. The most affected pathways were related to the metabolism of organic acids (all diseases), amino acids (all diseases), and glycine and its conjugates (in PA); the transsulfuration pathway; oxidative processes; and neurosteroid hormones (in cblC). The untargeted metabolomics study highlighted the presence of significant differences between the three diseases, pointing to the most relevant contrast in the cblC profile compared to MMA and PA. Some new biomarkers were proposed for PA, while novel data regarding the alterations of steroid hormone profiles and biomarkers of oxidative stress were obtained for cblC disease. The elevation of neurosteroids in cblC may indicate a potential connection with the development of ocular and neuronal deterioration.

## 1. Introduction

Propionic academia (PA) and methylmalonic academia (MMA) belong to the category of “classic” organic acidemias, a group of inherited diseases that result from the deficient activity of enzymes involved in the metabolism of propionate, whose precursors are the amino acids valine, isoleucine, threonine, and methionine, along with odd-chain fatty acids, cholesterol side chains, and propionate produced by the gut flora [1]. PA is caused by mutations in the PCCA and PCCB genes, coding for the two subunits of propionyl-CoA carboxylase, while MMA is caused by mutations in the MUT gene coding for the synthesis of methylmalonyl-CoA mutase [1]. A third disease, cobalamin C defect (cblC), due to mutations in the MMACHC gene, which encodes for a protein involved in intracellular vitamin B12 metabolism, impairs the synthesis of adenosylcobalamin and methylcobalamin, the cofactors of methylmalonyl-CoA mutase and of methionine synthase [2]. These three diseases, sharing a deficiency in propionate metabolism, produce various phenotypes and complex downstream metabolic effects. Clinically, PA and MMA can present with recurrent attacks of metabolic decompensation, mainly characterized by acidosis, hyperammonemia, ketosis, and elevated blood lactate. Acute neurological symptoms are common and can range from mild encephalopathy to metabolic strokes involving the basal ganglia [1]. Chronic complications of both diseases include a failure to thrive, developmental delays, movement disorder, deafness, and optic atrophy [1]. PA can additionally present with cardiomyopathy and QT prolongation, while MMA is also associated with chronic kidney disease leading to progressive renal failure [1]. Patients with cblC present the major involvement of the central nervous system (CNS) with neurocognitive impairment and develop a multisystem disease with progressive visual dysfunction, atypical hemolytic–uremic syndrome, and pulmonary hypertension [3].

Biochemically, PA, MMA, and cblC accumulate “primary” metabolites in the body fluids, which are derived from disruptions of metabolic pathways. These include propionyl-carnitine, 3-hydroxypropionic acid, and 2-methylcitric acid, all derived from un-metabolized propionyl-CoA [4]. As a consequence of the deficient activity of methylmalonyl-CoA mutase, MMA and cblC share the “primary” accumulation of methylmalonic acid, while a defect in methionine synthase in cblC causes the accumulation of homocysteine and the reduced synthesis of methionine [2]. Changes in in the levels of biomarkers, such as 2-methylcitric acid, propionylcarnitine, and methylmalonic acid, have demonstrated clinical relevance in patients with PA and MMA [4,5]. Moreover, the elevated homocysteine in cblC causes diffuse macroangiopathic damage, which is responsible for many of the clinical manifestations [3]. Abnormal circulating levels of ammonia, lactic acid, glycine, glutamine, and FGF21, which represent the “secondary” impact of the underlying genetic defect on metabolic and cellular pathways, are detectable in PA and MMA and only very rarely in cblC [3,4,5].

These abnormalities, detectable in a patient’s body fluids through targeted metabolomics analyses, represent the biochemical hallmarks that characterize the three disorders and have been validated for diagnosis and to evaluate the response to therapeutic interventions, including liver or liver and kidney transplantation in MMA [2,4,6,7]. However, there is still a need to determine whether the discovery of other metabolites can provide novel insights enabling us to understand the mechanisms that underlie the pathophysiology and aberrant processes of the diseases in relation to their clinical manifestations. For this purpose, thanks to rapid developments in bio-informatics and analytical technologies, it has been possible to expand metabolomics from targeted to untargeted analyses. The evolution of analytical platforms based on high-resolution mass spectrometry (HR-UHPLC-MS/MS) has allowed the simultaneous measurement of the broadest range of metabolites without a priori knowledge of the metabolome, in contrast with targeted metabolomics, in which metabolites are analyzed on the basis of a priori information [8]. The power of untargeted metabolomics therefore lies in its potential to broaden our understanding of the disease’s biochemistry, identifying new biomarkers and providing more precise disease categorization in relation to treatment modalities and responses.

Considering the relatively recent introduction of untargeted metabolomics in the area of inborn errors of metabolism [9], only a few studies have so far addressed MMA and PA, showing the impairment of serine, thiol, and propionate metabolism [10,11]; the dysregulation of the tricarboxylic acid cycle [12]; and changes in some known biomarkers in relation to the patient’s status [13]. As for cblC, untargeted metabolomics was applied as a screening tool with the aim of selecting diagnostic biomarkers and confirming methylmalonic and 2-methylcitric acids, propionylcarnitine, C4DC-carnitine, homocysteine, and methionine as the most reliable in discriminating cblC from other metabolic diseases [14,15].

In this work, we studied and compared the untargeted urinary metabolomic profiles of MMA, PA, and cblC to determine comprehensive disease-specific biomarker signatures, aiming at providing novel insights into the biological and pathophysiological differences between the three diseases.

## 2. Experimental Design

### Patients

Urine samples were obtained from 21 patients attending the Division of Metabolic Diseases at the Bambino Gesù Children’s Hospital in Rome. All patients had a confirmed biochemical and molecular diagnosis of MMA, PA, or cblC. Each disease group was composed of 7 patients of both sexes, and the age was matched among groups spanning the following ranges in years: MMA, 0.3–22; PA, 0.4–20.4; cblC, 0.4–17.1. All patients within the same disease group were treated with standard therapy, which included natural protein restriction, supplemented with an amino acid mixture free of offending precursors (MMA, PA), oral carnitine (MMA, PA, cblC), parenteral hydroxocobalamin (cblC), and oral betaine (cblC) therapy. Clinically, all patients presented with a severe phenotype, characterized by the early onset of the disease and by multiorgan involvement. Two out of 3 patients identified via newborn screening were already symptomatic. At the time of urine collection for untargeted metabolomics analysis, none of the 21 patients had been subjected to organ transplantation or dialysis. During the disease course, patients PA 1, PA 3, PA 5, PA 6, MMA 2, and MMA 5 were treated with liver transplantation and patients MMA 1, MMA 4, and MMA 7 with combined liver and kidney transplantation, which further confirmed the severity of the disease phenotype. Table 1 describes in more detail the clinical phenotypes of each patient.

This research was not a clinical trial and the procedure was in accordance with the Declaration of Helsinki of the World Medical Association. The study was approved by the Ethical Committee of the Bambino Gesù Children’s Hospital (n. 2119_OPBG_2020) and informed consent was obtained from all patients/caregivers.

## 3. Procedure

### 3.1. Sample Preparation

Urine samples were collected during scheduled follow-up visits and immediately frozen at −80 °C prior to analysis. Sample preparation consisted of mixing 200 µL of urine with 200 µL of organic solvent (acetonitrile and methanol 3:1) for HILIC analysis or with 200 µL of water for the reverse-phase UHPLC experiment. After 20 sec vortexing and 10 min centrifugation at 13,000 rpm, the supernatants were transferred into UHPLC vials for injection. A pooled sample was prepared by mixing equal aliquots of all urine samples. This pool underwent the same preparation steps and was used for data-dependent MS2 acquisition and as quality controls (QC).

### 3.2. UHPLC and HRMS

Ultra-high-performance chromatographic separation was executed on an Ultimate 3000 (Thermo Fisher Scientific, Waltham, MA, USA) system under reverse-phase and HILIC conditions. The reverse-phase experiment was carried on a Luna Omega C18 column (100 × 2.1 mm, 1.6 µm PS, Phenomenex, Torrance, CA, USA) with mobile phases A composed of water with 0.1% formic acid and B composed of acetonitrile with 0.1% formic acid. HILIC chromatographic separation was run on an Accucore-150-Amide column (100 × 2.1 mm, 2.6 µm, Thermo Fisher Scientific, Waltham, MA, USA) with mobile phases A (95% acetonitrile, 0.1% acetic acid, 10 mM ammonium acetate) and B (50% acetonitrile, 0.1% acetic acid, 10 mM ammonium acetate). The chromatographic gradients and instrument sets are available in Tables S1 and S2 in the Supplementary Materials. All samples were injected three times as technical replicates in random order. QC samples for peak area and RT correction were analyzed repeatedly across the whole batch afterwards.

The UHPLC system was coupled with a Q Exactive mass spectrometer (Thermo Fisher Scientific, Waltham, MA, USA) scanning in full MS and dd-MS2 modes. The full-scan MS experiment was set to a range of 70–1050 m/z with a 140,000 resolution, 3 × 10^6^ AGC target, and maximum IT 100 ms. dd-MS2 mode, used only for pool sample acquisition, was set at a 17,500 resolution, 2 × 10^4^ AGC target, maximum IT 35 ms, TopN 12, isolation window 1.5 m/z, and dynamic exclusion 2.5 s. The features were fragmented with three different normalized collision energy levels set to 20, 35, and 50 V. The source ionization parameters were spray voltage ± 3.5 kV; capillary temperature 380 °C; sheath gas 60; auxiliary gas 20; S-Lens level 50. The data were acquired in both positive and negative mode independently. Calibration was performed before each analysis using calibration mixes (Piercenet, Thermo Fisher Scientific, Rockford, IL, USA) to minimize the ppm error of the intact mass.

### 3.3. Metabolomic Data Analysis

The raw data were processed on the Compound Discoverer 3.3 (Thermo Fisher Scientific, Waltham, MA, USA) software for deconvolution, compound identification, and statistical analysis. To account for variations in the sample dilution factors, the acquired data were normalized with the Constant Sum algorithm integrated into Compound Discoverer 3.3. The time-dependent batch effects were corrected with the use of QC samples, implemented to build the linear regression model describing the variability in the chromatographic peak area of each m/z at different acquisition times. After the alignment and grouping of chromatographic peaks with the same molecular weight and retention time, the background features were removed from the results table. Subsequently, the features were statistically analyzed to identify characteristic compounds discriminating each disease. Principal component analysis (PCA) and volcano plots were applied to highlight the differences between the groups. Data annotation was performed by searching matching compounds through a home-made library (90 metabolites) and the ChemSpider, Metabolika, and mzCloud databases. All proposed metabolite structures were verified via the FISH score, which was assigned by comparing the experimental and in silico fragmentation patterns. PCA and box-and-whisker plots were generated in Compound Discoverer 3.3, and heatmap creation and pathway enrichment analysis were performed with the MetaboAnalyst 6.0 web platform. Pearson correlation coefficients (r) were calculated with Excel Microsoft 2016.

## 4. Results

After spectral deconvolution, alignment, QC, and background correction, the data from HILIC, in both polarity modes, and from the reverse phase (C18), only in negative mode, were considered for subsequent elaborations. The data from the reverse-phase C18 positive mode were discarded because they did not fulfill the Compound Discoverer 3.3 quality criteria.

**Figure 1:**
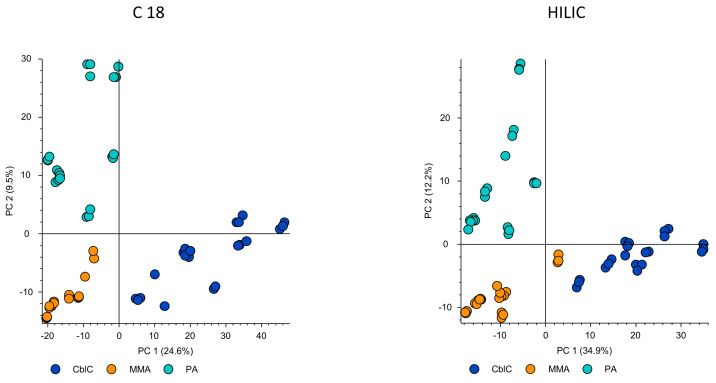
PCA obtained from untargeted metabolomics data acquired by C18 and HILIC columns in negative ionization mode. Both experimental conditions demonstrate the major separation of the cblC group and the closer similarity between the MMA and PA groups.

The untargeted metabolomics data showed the net differences between the three diseases. The PCA plots obtained from the different chromatographic conditions demonstrated a coherent picture pointing to the most characteristic metabolomic profile of the cblC group, which resulted in the highest separation from the other diseases (PC1 > 24.6%), while the profiles of MMA and PA were more similar between them (PC2 > 9.5%); see Figure 1.

### 4.1. Significantly Different Metabolites

The volcano plots, set to *p* < 0.05, log_2_FC > |1|, revealed 530 characteristic features for MMA, 450 for PA, and 1700 for cblC. Only some of the characteristic features were annotated. Additionally, we selected a group of features with the molecular formula CxHyNO, likely belonging to the same chemical class, that were significantly increased in PA. The most relevant findings are summarized in Table 2, while the whole list of putative annotations is given in Supplementary Table S3.

**Figure 2:**
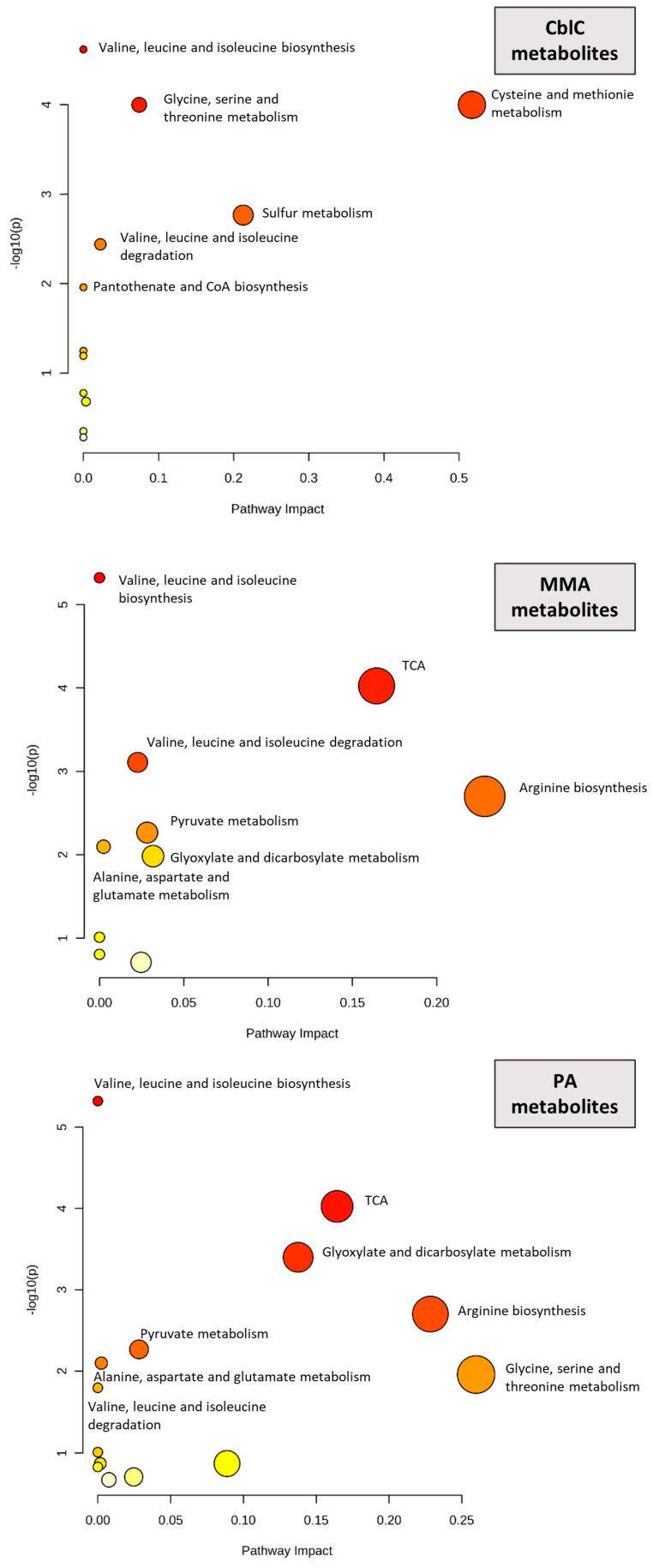
Pathway enrichment analysis. Node size (pathway impact) corresponds to the relative number and position of matched metabolites in the selected pathway; colors, varying from yellow to red, indicate the different levels of significance. The named pathways include only those with *p* < 0.05.

The significantly increased metabolites in each disease were associated with known KEGG metabolic pathways. Figure 2 demonstrates the main pathways involved in the three metabolic diseases.

All diseases shared the involvement of valine, leucine, and isoleucine biosynthesis and degradation pathways. CblC and PA showed the common involvement of glycine, serine, and threonine metabolism-related pathways, while PA and MMA shared pathways related to the Krebs cycle (TCA), pyruvate metabolism, glyoxylate and dicarboxylate metabolism, and arginine biosynthesis, along with alanine, aspartate, and glutamate metabolism. The main pathways involved in cblC and not in MMA and PA included cysteine and methionine metabolism, sulfur metabolism, and pantothenate and CoA biosynthesis.

#### 4.1.1. Organic Acids and Ketones

**Figure 3:**
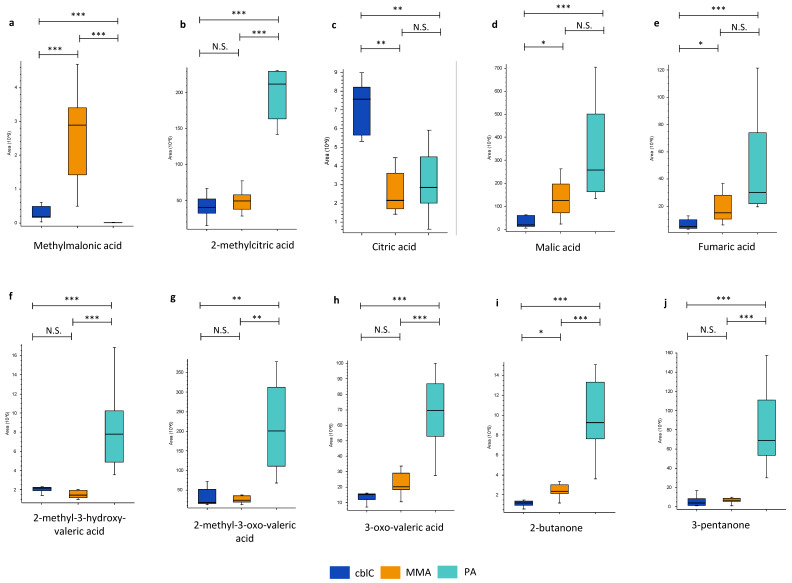
Conventional organic acid biomarkers of MMA, PA, and cblC: (**a**) methylmalonic acid, (**b**) 2-methylcitric acid, (**f**) 2-methyl-3-hydroxy-valeric acid, (**g**) 2-methyl-oxo-valeric acid, (**h**) 3-oxo-valeric acid; Krebs cycle organic acids (**c**) citric acid, (**d**) malic acid, (**e**) fumaric acid; and ketones (**i**) 2-butanone, (**j**) 3-pentanone significantly changed between three acidemias. *—*p*-value < 0.05; **—*p*-value < 0.01; ***—*p*-value < 0.001; N.S.—non-significant.

The untargeted metabolomics study confirmed the presence of known primary disease-related biomarkers, revealing a significant increase in methylmalonic acid in MMA and in cblC compared to PA, while methylcitric acid was significantly increased in PA compared to MMA and cblC (Table 2 and Figure 3). The most discriminant organic acids and ketones in PA were 3-pentanone, 2-methyl-3-oxo-valeric acid, 2-methyl-3-hydroxy-valeric acid, 2-butanone, and 3-oxo-valeric acid (Table 2 and Figure 3). Moreover, 3-hydroxypropionic acid was also increased in PA, but, due to the low quality of the chromatographic peak integration caused by the presence of coeluting isomers, it was not considered for quantitative estimation.

The profiles of the organic acids related to the Krebs cycle showed significantly reduced levels of citric and isocitric acids (not separated chromatographically) along with increased malic and fumaric acids in MMA and PA compared to cblC; see Figure 3. Other organic acids related to the TCA cycle did not show differences between the three disease groups.

A feature with an m/z and fragmentation pattern putatively corresponding to propionic acid was highly increased in the MMA and cblC groups with respect to PA (Table 2, Supplementary Figure S1a). However, its levels were strongly correlated across all samples (r > 0.9) with methylmalonic acid (Supplement Figure S1b), allowing us to consider this compound as propionic acid derived from the in-source fragmentation of methylmalonic acid.

#### 4.1.2. Amino Acids and Peptides

**Figure 4:**
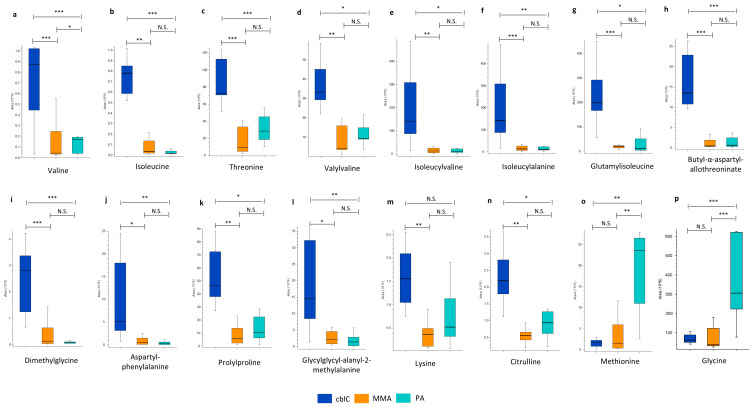
Significantly changed amino acids: (**a**) valine, (**b**) isoleucine, (**c**) threonine, (**m**) lysine; and small peptides (**d**) valylvaline, (**e**) isoleucylvaline, (**f**) isoleucylalanine, (**g**) glutamylisoleucine, (**h**) butyl-alpha-aspartyl-allothreoninate, (**j**) aspartyl-phenylalanine, (**k**) prolylproline, (**l**) glycylglycyl-alanyl-2-methylalanine increased in cblC may be a result of dietary differences between groups. Significantly changed levels of (**i**) dimethylglycine, (**n**) citrulline, (**o**) methionine, and (**p**) glycine reflect the involvement of different metabolic pathways in the diseases. *—*p*-value < 0.05; **—*p*-value < 0.01; ***—*p*-value < 0.001; N.S.—non-significant.

Several free amino acids and small peptides demonstrated significantly different levels between the three disease groups (Table 2 and Figure 4).

Consistently with dietary restrictions, MMA and PA showed reduced levels of valine, isoleucine, and threonine and of di- and tri-peptides composed of branched-chain amino acids and/or threonine (i.e., isoleucylalanine, glutamylisoleucine, valylvaline, isoleucylvaline, butyl-alpha-aspartyl-allothreoninate). In addition, other amino acids and peptides (i.e., dimethylglycine, aspartylphenylalanine, prolylproline, glycylglycyl-alanyl-2-methylalanine, lysine, and citrulline) were relatively higher in cblC compared to MMA and PA.

Methionine was significantly reduced in cblC, and also in MMA, compared to PA (Figure 4o). The glycine level in PA was increased up to 30- and 20-fold compared to MMA and cblC, respectively (Figure 4p). The levels of glycine and methionine had no significant differences between MMA and cblC.

#### 4.1.3. Glycine and Carnitine Conjugates

**Figure 5:**
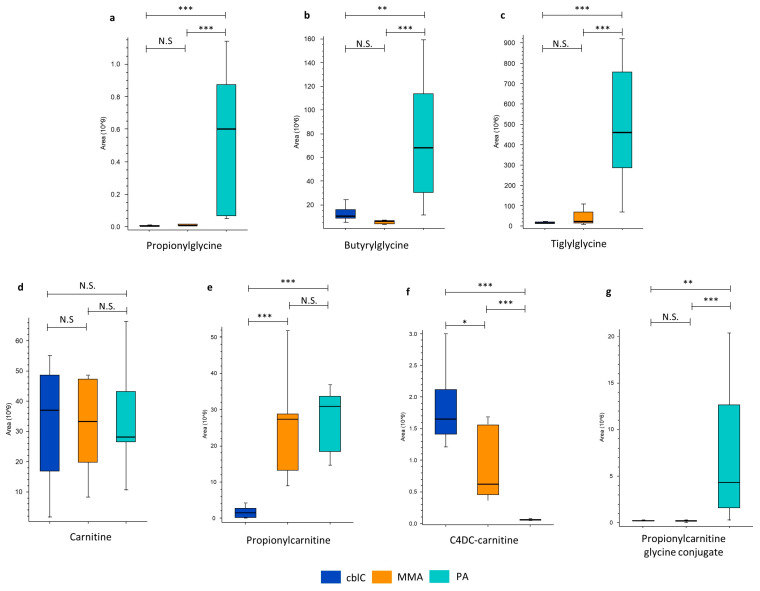
Glycine and carnitine conjugates. Glycine conjugates increased only in PA group: (**a**) propionylglycine, (**b**) butyrylglycine, (**c**) tiglylglycine, (**g**) glycine conjugate of propionylcarnitine; carnitine conjugates increased in MMA and PA: (**e**) propionylcarnitine; and in MMA and cblC: (**f**) c4DC-carnitine; (**d**) free carnitine had no differences between groups. *—*p*-value < 0.05; **—*p*-value < 0.01; ***—*p*-value < 0.001; N.S.—non-significant.

Three glycine esters, propionylglycine, butyrylglycine, and tiglylglycine, were markedly increased in PA compared to MMA and cblC (Table 1 and Figure 5).

In particular, propionylglycine was increased up to 100-fold and 60-fold with respect to cblC and MMA. Propionylglycine (r > 0.9) and butyrylglycine (r > 0.7) were positively correlated with glycine and between them (r > 0.7) The levels of other glycine conjugates, like N-acetylglycine, glutamylglycine, isovalerylglycine, isocaprolylglycine, hexanoylglycine, and hippuric acid, had no differences between the three disease groups.

Different from glycine and its esters, free carnitine had similar levels in cblC, PA, and MMA, while propionylcarnitine was equally increased in PA and MMA compared to cblC (Table 1 and Figure 5e). The C4DC-carnitine levels in cblC exceeded those in MMA and PA (Table 1 and Figure 5f). Interestingly, a metabolite corresponding to propionylcarnitine conjugated to glycine was selectively elevated only in PA and not in MMA or cblC (Table 1 and Figure 5g). The proposed putative structure of this double carnitine/glycine conjugate produced the in silico fragmentation pattern corresponding to the experimental MS/MS data at 93.7% (Supplementary Figure S2a,b).

#### 4.1.4. Transsulfuration Pathway Metabolites

**Figure 6:**
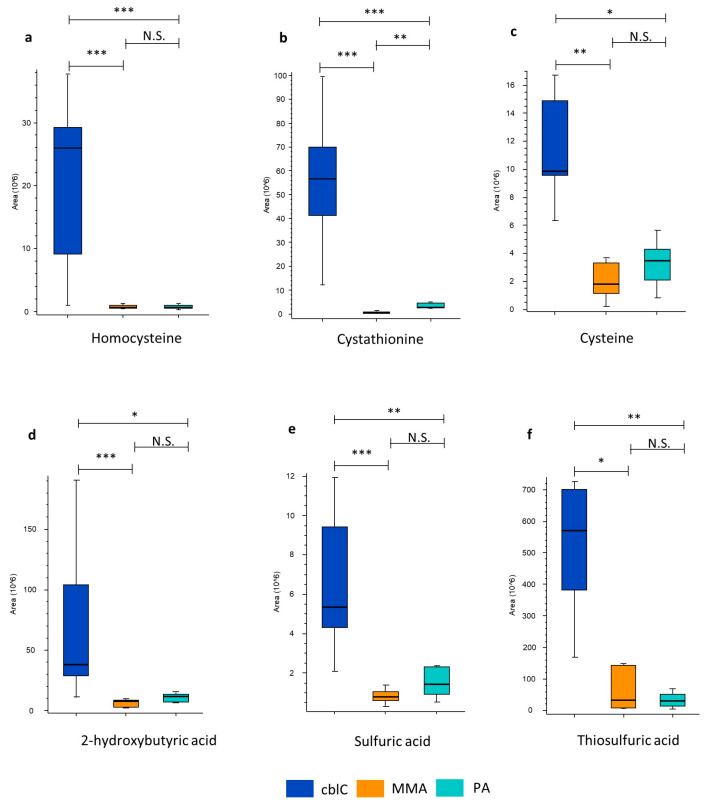
Increased (**a**) homocysteine and transsulfuration pathway metabolites in cblC: (**b**) cystathionine, (**c**) cysteine, (**d**) 2-hydroxybyryric acid; and oxidized sulfur-containing anions: (**e**) sulfuric and (**f**) thiosulfuric acid. *—*p*-value < 0.05; **—*p*-value < 0.01; ***—*p*-value < 0.001; N.S.—non-significant.

Several metabolites belonging to the transsulfuration pathway, including homocysteine, cystathionine, cysteine, and sulfuric and thiosulfuric acids, were selectively increased in cblC (Table 1 and Figure 6).

Among these metabolites, cystathionine and homocysteine demonstrated the most relevant and significant increase, reaching 78-fold and 39-fold changes compared to the levels in MMA and PA. Moreover, 2-hydroxybutyric acid, putatively derived from alpha-ketobutyric acid, was also elevated in cblC.

#### 4.1.5. New Characteristic Compounds of cblC Disease

##### Biomarkers of Oxidative Damage

**Figure 7:**
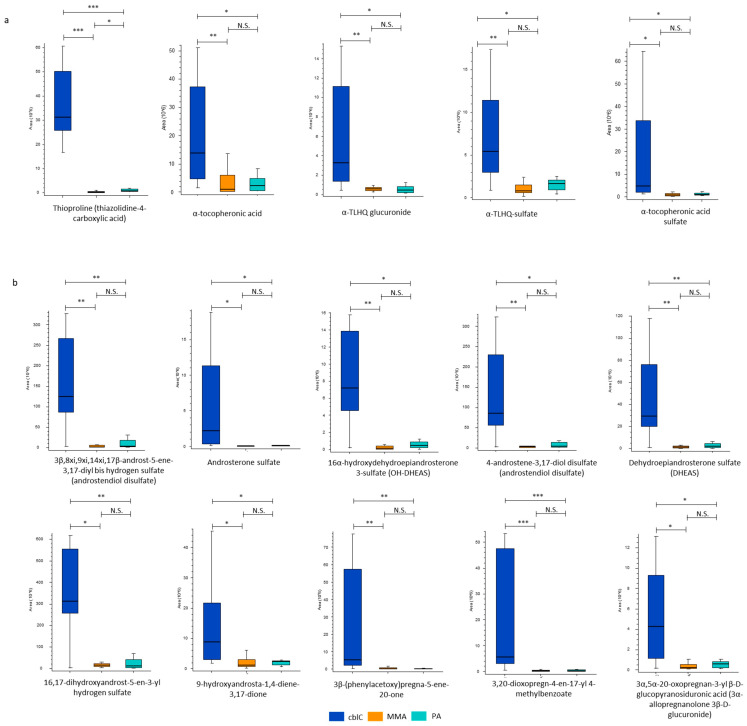
New characteristic compounds of cblC disease. (**a**) Oxidative stress biomarkers. (**b**) Steroid hormones with putative annotations belonging to androstane core class, namely 3β,8xi,9xi,14xi,17β-androst-5-ene-3,17-diyl bis hydrogen sulfate, androsterone sulfate, 16α-hydroxydehydroepiandrosterone-3-sulfate, 4-androstene-3β,17β-diol disulfate, dehydroepiandrosterone sulfate, 16,17-dihydroxyandrost-5-en-3-yl hydrogen sulfate, 9-hydroxyandrosta-1,4-diene-3,17-dione, and pregnane core class, namely 3β-(phenylacetoxy)pregna-5-ene-20-one; 3,20-dioxopregn-4-en-17-yl 4-methylbenzoate; 3α,5α-20-oxopregnan-3-yl beta-D-glucopyranosiduronic acid. *—*p*-value < 0.05; **—*p*-value < 0.01; ***—*p*-value < 0.001; N.S.—non-significant.

Urine obtained from cblC patients showed a major increase in compounds related to oxidative stress; see Figure 7a. These included thioproline, a sulfur-containing non-proteinogenic amino acid, formed by the condensation between cysteine and formaldehyde, which was increased up to 96-fold and 45-fold compared to MMA and PA, respectively. Other metabolites, significantly elevated in the cblC group, were derived from the oxidation of vitamin E by peroxyl radicals. These included α-tocopheronic acid, α-tocopheronolactone hydroquinone (α-TLHQ) glucuronide, α-TLHQ sulfate, and α-tocopheronic acid sulfate.

##### Steroid Hormones

The cblC group was characterized by the increased levels of steroid compounds with an androstane or pregnane core. The putative annotations corresponding to derivatives of dehydroepiandrosterone (DHEA), pregnanolone, and others are given in Figure 7b. Six out of 10 compounds were in sulfate form and one was a glucuronic acid conjugate. The urinary levels of these steroids in the cblC group largely exceeded those in MMA and in PA.

#### 4.1.6. Non-Annotated Metabolites CxHyNO in PA

**Figure 8:**
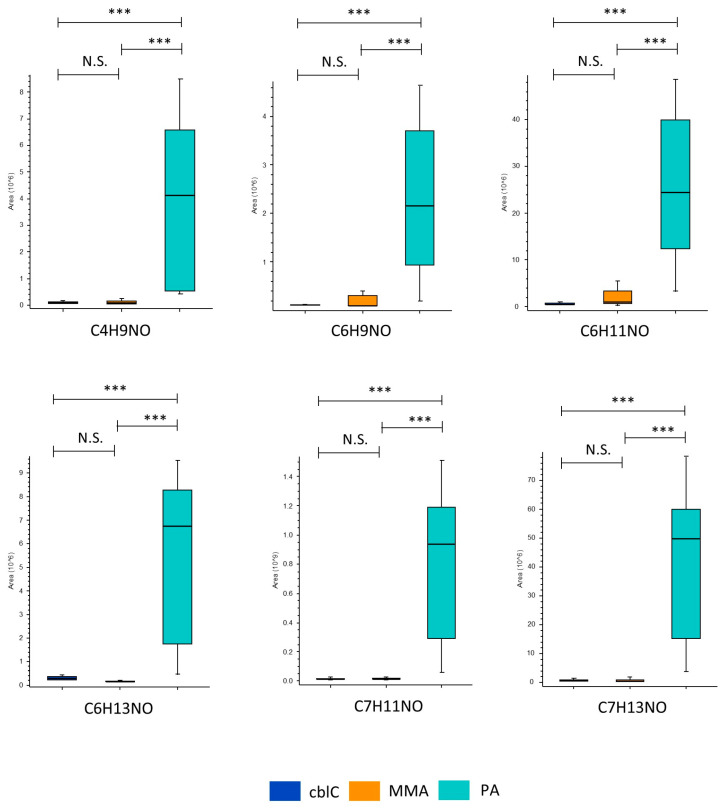
The CxHyNO features significantly increased in PA. ***—*p*-value < 0.001; N.S.—non-significant.

A group of six non-annotated metabolites, having a CxHyNO elemental composition with carbon atom lengths C4-C7, were significantly increased in the group of PA. C7H13NO was the most increased one, with an up to 142-fold change with respect to MMA and cblC. Other features were C7H11NO, C6H13NO, C6H11NO, C6H9NO, and C4H9NO (Table 1 and Figure 8).

#### 4.1.7. The Most Dysregulated Non-Annotated Features

**Figure 9:**
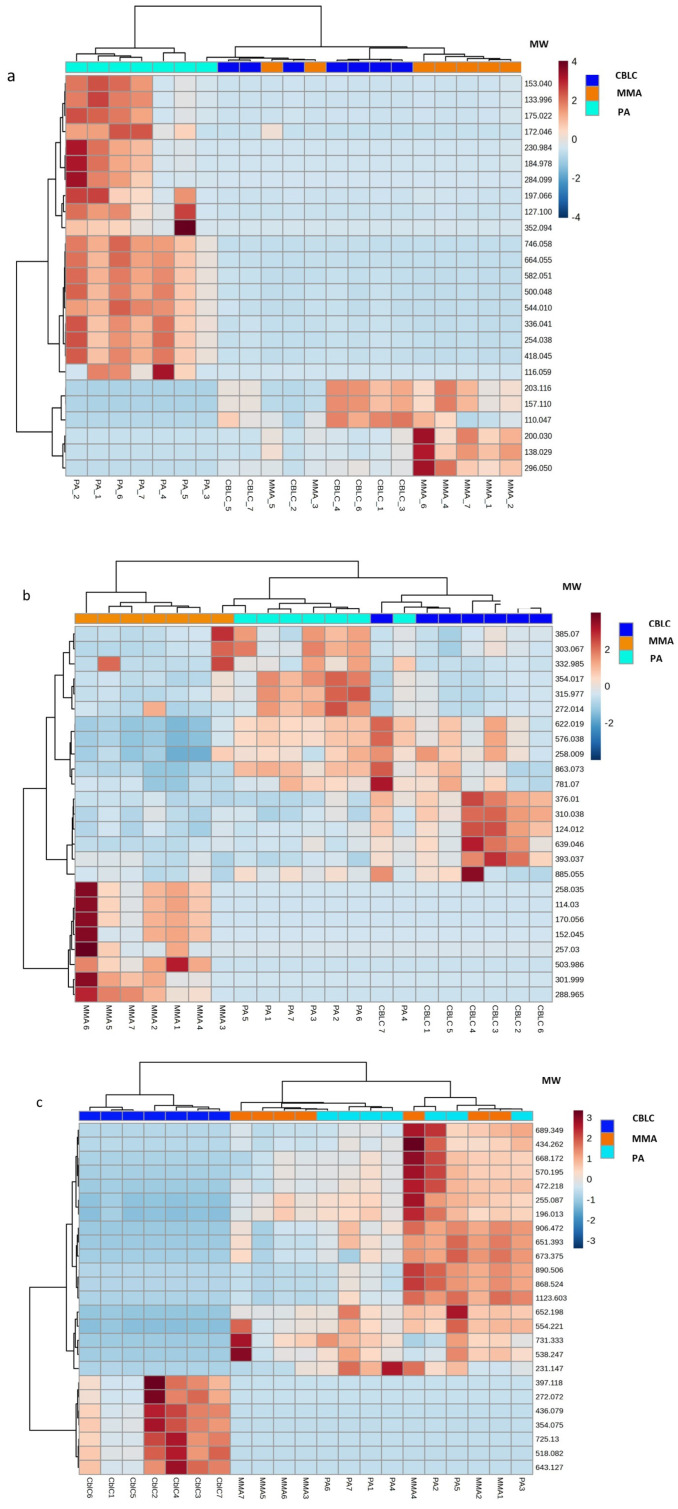
(**a**) The heatmap based on the 25 most up-/down-regulated features in PA (*p* < 0.001); (**b**) the heatmap based on the 25 most up-/down-regulated features in MMA (*p* < 0.001); (**c**) the heatmap based on the 25 most up-/down-regulated features in cblC (*p* < 0.001).

The 25 most up- and down-regulated non-annotated features for each disease (*p* < 0.001) are listed in Supplementary Tables S4–S6. Heatmaps with the hierarchical clustering of these features are shown in Figure 9.

The PA group was characterized by elevated levels of 19 features with molecular weights of 116–746 Da, with eight of them coherently increased across all PA samples. Six features with lower molecular weights (110–296 Da) were decreased in all PA samples with respect to the cblC and MMA groups (Figure 9a). MMA group was characterized by lower levels of 17 species with molecular weights of 124–885 Da. A single feature, with a molecular weight of 288.965 Da, was constantly increased in all MMA samples (Figure 9b). In the cblC group, 18 out of 25 features were significantly decreased compared to MMA and PA. These features generally had high molecular weights (up to 1123 Da) and demonstrated constantly decreased levels across all cblC samples (Figure 9c).

## 5. Discussion

In this study, we performed a comparison of the untargeted metabolomic profiles in the urine of patients with three organic acidemias, MMA, PA, and cblC, sharing the involvement of the propionate pathway. Our results highlighted a clear distinction between the three diseases, showing the relative differences in the levels of known biomarkers, and described new characteristic metabolites, allowing a more profound understanding and knowledge of the (biochemical) phenotypic disease characteristics and pathophysiology.

The pathway enrichment analysis indicated the involvement of branched chain amino acid metabolism in all diseases, pointing at major perturbations affecting the Krebs cycle in PA and MMA. The specificities of cblC, contributing to the highest separation from the other diseases, were related not only to sulfur metabolism but also to other novel pathways.

Relative quantification in untargeted metabolomics applied to urine requires the normalization of the data. For this purpose, different normalization strategies were developed to reduce the measurement errors [16,17,18], but no consensus has been so far reached on the most suitable approach for urine standardization [17,19,20]. Normalization to creatinine is the most used method in targeted metabolomic analyses [21,22]; however, MMA and PA may affect creatine metabolism, leading to reduced creatinine formation [23,24,25,26]. For this reason, for our study, we applied the Constant Sum algorithm for post-acquisition normalization.

According to targeted metabolomics analyses, the main shared biomarkers of PA, MMA, and cblC are 3-hydroxypropionic and 2-methylcitric acids [2,3,4,5]. In line with this, the results of our untargeted metabolomics study in urine showed a major increase in 2-methylcitric acid in PA compared to MMA and cblC. As for methylmalonic acid, shared by MMA and cblC [27], it was predominantly increased in MMA. Unexpectedly, and similarly to a previous report [13], we identified a metabolite with a molecular weight and fragmentation pattern corresponding to propionic acid, highly increased in MMA and cblC but not in PA. However, the levels of this compound and methylmalonic acid were strongly correlated across the samples and a more accurate evaluation allowed us to demonstrate that the presumed “propionic acid” was an artifact, derived from the in-source fragmentation of methylmalonic acid molecules due to the loss of CO_2_ [28,29].

As for other biomarkers, a selective increase in 2-methyl-3-oxo-valeric acid, 2-methyl-3-hydroxy-valeric acid, 3-oxo-valeric acid, 3-pentanone, and 2-butanone was detected in PA. Oxo-valeric acid-related metabolites are derived from the self-condensation of propionyl-CoA, 3-pentanone may result from the decarboxylation of 2-methyl-3-oxo-valeric acid [30], while 2-butanone is a product of methylacetoacetate decarboxylation in the catabolic pathway of isoleucine [31]. From a clinical perspective, the presence of alpha- and beta-ketoaciduria may explain the frequent observation in PA patients of positive ketones in urine, as detected by commercial strips (testing for acetoacetate through a nitroprusside reaction), which does not correspond to the presence of ketonemia (elevated beta-hydroxybutyrate levels in blood; personal observation of Carlo Dionisi-Vici).

Focusing on TCA metabolites, the citrate levels in MMA and PA were significantly reduced compared to cblC, likely reflecting the major accumulation of propionyl-CoA, which competes with acetyl-CoA as a substrate for citrate synthase, generating 2-methylcitrate after condensation with oxaloacetate [32]. The accumulation of 2-methylcitrate and of other “toxic” compounds derived from methylmalonyl-CoA and propionyl-CoA metabolism may impact the TCA cycle and the urea cycle, leading to a shortage of TCA intermediates, to mitochondrial dysfunction, and to hyperammonemia [32,33]. In accordance with this, increases in the malate, fumarate, and lower citrulline levels were found in PA and MMA samples. Perturbations of these metabolites, which are known to be related to mitochondrial dysfunction [34,35,36,37], may therefore reflect and explain the greater mitochondrial involvement in PA and MMA compared to cblC.

MMA and PA are both characterized by abnormal glycine metabolism [38]. Our study, while confirming this perturbation, showed that the levels of glycine in PA were about 30 times higher than those seen in MMA. This difference highlights that different (patho)mechanisms may lead to increased glycine in the two diseases. In MMA, a widespread posttranslational modification, methylmalonylation, inhibits the glycine cleavage pathway [38]. In PA, as shown by ^13^C-flux experiments in PCCA-null cells, the increased de novo synthesis of glycine occurs from serine [10]. Remarkably, we found that the increased glycine levels in PA significantly correlated with an increase in its esters, propionylglycine and butyrylglycine, further confirming that PA affects glycine metabolism to a greater extent than in MMA. Taken together, these findings seem to indicate that the increased glycine synthesis in PA may be considered as a “defense” mechanism, aimed to buffer the toxic accumulation of propionyl-CoA, through the action of glycine-N-acylase, as seen in isovaleric acidemia [39,40,41]. Consistently with this, we identified a novel glycine conjugate of propionyl-carnitine that was significantly elevated in PA.

The analysis of carnitine derivatives, reflecting the buffering action of carnitine-acetyltransferase on the mitochondrial excess of propionyl-CoA, showed less pronounced differences than those seen for glycine esters. Propionylcarnitine was equally increased in PA and MMA in comparison to cblC, while C4DC-carnitine, combining methylmalonyl- and succinyl-carnitines [42], was higher in cblC than in MMA.

This study showed significant differences in the excretion of amino acids and small peptides in cblC in comparison to PA and MMA. The relative elevation in cblC of the majority of these compounds is likely the consequence of the dietary differences between the three diseases. All cblC patients were on a free diet, while PA and MMA patients were treated with a protein-restricted diet to limit the intake of propionic acid precursors [6]. Accordingly, the levels of isoleucine, valine, threonine, and related small peptides were significantly reduced in PA and MMA. On the other hand, a significant reduction in methionine was detected in cblC, reflecting the deficient activity of methionine synthase as a consequence of the reduced availability of its cofactor methylcobalamin [2]. The large increase in dimethylglycine in cblC samples is consistent with the activation of the folate-independent homocysteine remethylation pathway through the action of betaine therapy [2].

As expected, increased levels of metabolites linked to the transsulfuration pathway were exclusively detected in the cblC group, as also reported by previous researchers [43,44]. The elevated levels of thiosulfate and sulfate ions indicate that, among the pathways of homocysteine and cysteine degradation, the most active would be the one related to H_2_S production, which is further oxidized to sulfates [45,46].

A common characteristic of MMA, PA, and cblC is elevated oxidative stress. Previous studies demonstrated the presence of reactive oxygen species and oxidative damage in these diseases [47,48,49,50,51]. In addition, cblC lymphocytes were characterized by an imbalance between the reduced/oxidized forms of glutathione [52]. The comparison of the metabolomic profiles between the three diseases allowed us to distinguish some specific pathways of oxidative stress that were characteristic only for cblC, rather than for MMA and PA. We identified oxidized forms of vitamin E metabolites, such as α-TLHQ, α-tocopheronic acid, and their conjugates. These molecules have been associated with multiple diseases characterized by lipid peroxidation and have been defined as the urinary biomarkers of oxidative stress [53,54,55]. Another specific indicator of oxidative stress damage that was uniquely detected in cblC patients was thioproline, which is related to formaldehyde metabolism. Thioproline is generated in oxidant-exposed cells and exerts a protective role through sacrificial oxidation and nitrile ion trapping [56]. Being a product of formaldehyde and cysteine conjugation, thioproline may be therefore considered as a scavenger of free endogenous formaldehyde released during demethylation and protein oxidation and from the metabolism of methanol, methylamine, and adrenaline [56]. The excess of formaldehyde in cblC may also be related to the oxidative breakdown of tetrahydrofolate, not employed for the folate-dependent methylation of homocysteine [57,58]. Besides conjugation with cysteine, other methods of formaldehyde buffering exist in mammals, such as enzymatic conversion through glutathione and adduction with histidine to form spinacine [59]. However, in our study, thioproline was the only formaldehyde adduct that was increased in cblC, while the levels of spinacine did not differ between the three diseases. Furthermore, we did not find features corresponding to glutathione-related products, such as S-hydroxymethyl-glutathione or S-formyl-glutathione [59]. Therefore, the elevated levels of thioproline in cblC may be explained by the preferential buffering of formaldehyde through its condensation with cysteine. The potential role of formaldehyde and thioproline in the pathophysiology of the cblC disease is worthy of further elucidation. Interestingly, both formaldehyde and thioproline accumulation have been associated with neurodegeneration [59,60].

The untargeted metabolomics study revealed a sharp increase in steroid hormones in the urine of cblC patients. The majority of these compounds belong to the class of “neurosteroids”, which can also have androgenic or estrogenic activity. Neurosteroids, synthetized in the brain or in the peripheral nervous system, can be transformed into sulfate or glucuronide conjugates and then excreted in the urine. The role of neurosteroids is very complex and lies in their ability to act as neurohormones, neuromodulators, neurotransmitters, and neurotrophic factors. They regulate arousal, sleep, learning, and social and sexual behaviors and are implicated in several (patho)physiological conditions, such as pain, stress, anxiety, and depression, but also in autism, neurodegenerative diseases (multiple sclerosis, Alzheimer’s disease, Parkinson’s disease), Niemann–Pick type C disease, and traumatic brain injury [61,62,63,64,65,66,67,68,69,70]. The presence of increased levels of neurosteroids in cblC patients may therefore indicate a potential connection with the peculiar neurological manifestations of this disease [2].

Furthermore, the involvement of steroid hormones in cblC may also reflect the characteristic disease-associated visual deterioration [2]. Specific receptors have been found in the visual system, including the lacrimal and meibomian glands, conjunctiva, cornea, lens, retina, and choroid [71]. Numerous studies on ocular diseases have revealed the net connection between steroid hormones and retinal, corneal, and optic nerve disorders such as cataracts, dry eye disease, glaucoma, age-related macular degeneration, retinitis pigmentosa, and central serous chorioretinopathy [71,72,73,74,75]. It has also been reported that both estrogens and androgens affect the retinal thickness, and their elevated levels were found in patients with chronic central serous chorioretinopathy [72,73,76].

Our study identified a potentially novel class of PA biomarkers characterized by the molecular formula CxHyNO with carbon lengths C4-C7. The similar elemental composition and close elution times may indicate the presence of the same functional group(s) derived from coherent metabolic pathways. These metabolites may belong to the class of amides (from the decarboxylation of acylglycines [77]), aminoaldehydes (from the oxidative degradation of polyamines [78]), nitroso-compounds, and/or acyl-pyrrolidine/pyrrolines.

It is well known that the annotation of features detected by untargeted metabolomics represents the greatest challenge. In this study, we selected the 25 most significantly increased/decreased non-annotated features for each disease and reported their accurate molecular weights to allow their confirmation and characterization in future metabolomics research on MMA, PA, or cblC.

**Figure 10:**
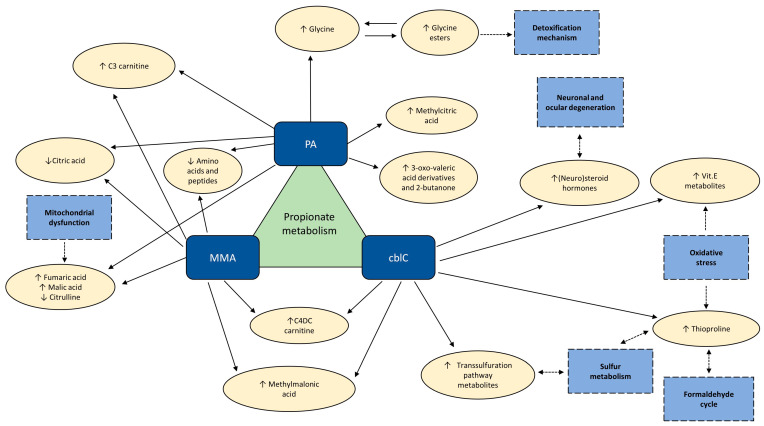
The most relevant findings from the relative comparison of the metabolomic profiles in PA, MMA, and cblC. ↑ (↓)—metabolites significantly increased (decreased) with respect to other two acidemias. Dashed boxes—physiological processes involving significantly changed metabolites.

The most relevant results of our study, demonstrating the different and common aspects in the metabolomic profiles of cblC, MMA, and PA acidemias, are summarized in Figure 10.

## 6. Conclusions

Our study allowed the discovery of new disease-related biomarkers like glycine conjugates of propionylcarnitine in PA and thioproline, vitamin E oxidation products, and steroid compounds in cblC, expanding the knowledge of novel potential pathophysiological mechanisms and demonstrating the importance of exploring, through untargeted metabolomics, the biochemical profiles of metabolic diseases.

## Figures and Tables

**Table 1:** Clinical data and symptoms of the patients.

Patient	Onset	Cardiac Involvement	Pancreatitis	Renal Disease	Visual Dysfunction	Epilepsy	Abnormal MRI	Developmental Delay/ Intellectual Disablity	Deafness	Age at Urine Sampling (Years)
PA 1	Neonatal	-	-	-	-	-	+	+	+	2.1
PA 2	7 months	+	-	-	-	-	+	+	-	9.9
PA 3	3 months	+	-	-	-	-	-	-	-	20.4
PA 4	Neonatal	+	-	-	-	-	-	+/-	+	15.2
PA 5	Neonatal (NBS symptomatic)	-	-	-	-	-	-	+/-	-	0.4
PA 6	Neonatal (NBS symptomatic)	-	-	-	-	-	+	+	-	0.8
PA 7	Neonatal	+	-	-	-	-	-	-	-	7.0
MMA 1	Neonatal	-	-	++	-	-	+/-	-	-	0.6
MMA 2	Neonatal	-	+	+/-	-	-	+/-	+	+	1.2
MMA 3	Neonatal	-	-	-	-	-	++	+	NA	10.6
MMA 4	17 months	-	-	++	+	-	-	-	-	20.6
MMA 5	NBS (asymptomatic)	-	-	-	-	-	-	-	-	0.3
MMA 6	Neonatal (NBS symptomatic)	-	-	-	-	-	-	-	NA	3.7
MMA 7	6 months	-	+	++	+	-	++	-	-	22.2
cblC 1	Neonatal (NBS symptomatic)	+	-	-	+	-	-	+/-	-	0.4
cblC 2	Neonatal	-	-	+	+	+	+	+	-	3.7
cblC 3	2 months	+	-	+	+	+	++	++	-	2.8
cblC 4	Neonatal	-	-	-	+	+	+	+	-	3.3
cblC 5	5 months	-	-	-	+	++	+	++	-	13.7
cblC 6	Neonatal	-	-	-/+	+	-	+	+/-	-	13.2
cblC 7	3 months	++	-	+	-	+	+/-	+/-	-	17.1

++ severe; + present; +/- mild; - absent; NA—not available; long-term follow-up—urine samples for metabolomics analysis were collected before transplantation.

**Table 2:** Significantly different metabolites in cblC, MMA, and PA and their relative fold-change values.

Metabolite	cblC/MMA	cblC/PA	PA/MMA	KEGG Pathway
Organic Acids and Ketones				
Citric and isocitric acids	3.3 **	2.5 **	ns	Tricarboxylic acid cycle (TCA) Glyoxylate and dicarboxylate metabolism Alanine, aspartate, and glutamate metabolism
2-Butanone	0.5 *	0.13 ***	3.9 ***	
Fumaric acid	0.3 *	0.2 ***	ns	TCA Arginine biosynthesis Pyruvate metabolism Alanine, aspartate, and glutamate metabolism
Malic acid	0.2 *	0.08 ***	ns	TCA Glyoxylate and dicarboxylate metabolism Pyruvate metabolism
Methylmalonic acid	0.06 ***	19 ***	0.003 ***	Valine, leucine, and isoleucine degradation Propanoate metabolism Pyrimidine metabolism
MW 74.036 (propionic acid isobar)	0.05 ***	12 ***	0.004 ***	
2-Methylcitric acid	ns	0.2 ***	4.3 ***	Propanoate metabolism
2-Methyl-3-hydroxy-valeric acid	ns	0.3 ***	5.4 ***	
3-Oxo-valeric acid	ns	0.21 ***	3.4 ***	
2-Methyl-3-oxo-valeric acid	ns	0.09 **	8.3 **	
3-Pentanone	ns	0.06 ***	10 ***	
Glycine and Carnitine Conjugates				
C4DC-carnitine	2.1 *	348 ***	0.006 ***	
Propionylcarnitine	0.06 ***	0.05 ***	ns	
Propionylglycine	ns	0.01 ***	60 ***	
Propionylcarinitine glycine conjugate	ns	0.05 **	23 ***	
Tiglylglycine	ns	0.07 ***	21 ***	
Butyrylglycine	ns	0.2 **	11 ***	
Amino Acids and Peptides				
Threonine	83 ***	36 ***	ns	Glycine, serine, and threonine metabolism Valine, leucine, and isoleucine biosynthesis
Butyl-α-aspartyl-allothreoninate	28 ***	20 ***	ns	
Isoleucine	24 **	50 ***	ns	Valine, leucine, and isoleucine biosynthesis Valine, leucine, and isoleucine degradation
Dimethylglycine	21 ***	36 ***	ns	Glycine, serine, and threonine metabolism
Isoleucylalanine	15 ***	3.4 **	ns	
Aspartylphenylalanine	9.6 *	23.4 **	ns	
Glutamylisoleucine	9.4 ***	19 *	ns	
Prolylproline	8.0 **	4.4 *	ns	
Valylvaline	7.7 **	3.6 *	ns	
Glycylglycyl-alanyl-2-methylalanine	6.7 *	11 **	ns	
Isoleucylvaline	5.0 **	4.9 **	ns	
Lysine	4.1 **	ns	ns	Biotin metabolism
Citrulline	3.9 **	2.3 *	ns	Arginine biosynthesis
Valine	3.8 ***	2.4 ***	1.6 *	Valine, leucine, and isoleucine biosynthesis Valine, leucine, and isoleucine degradation Pantothenate and CoA biosynthesis
Methionine	ns	0.07 **	16 **	Cysteine and methionine metabolism 2-Oxocarboxylic acid
Glycine	ns	0.04 ***	33 ***	Glycine, serine, and threonine metabolism Glyoxylate and dicarboxylate metabolism Glutathione metabolism Lipoic acid metabolism Porphyrin metabolism
Transsulfuration Pathway Metabolites				
Cystathionine	78 ***	20 ***	3.8 **	Glycine, serine, and threonine metabolism Cysteine and methionine metabolism
Homocysteine	37 ***	39 ***	ns	Cysteine and methionine metabolism
Thiosulfuric acid	17 *	18 **	ns	Sulfur metabolism
Cysteine	5.7 **	3.5 *	ns	Glycine, serine, and threonine metabolism Pantothenate and CoA biosynthesis Glutathione metabolism Thiamine metabolism
2-Hydroxybutyric acid	5.0 ***	3.2 *	ns	Propanoate metabolism
Sulfuric acid	4.2 ***	2.5 **	ns	Sulfur metabolism
Biomarkers of Oxidative Damage				
Thioproline	96 ***	45 ***	2.1 *	
α-Tocopheronic acid	14 **	5.9 *	ns	
α-TLHQ glucuronide	8.0 **	4.7 *	ns	
α-TLHQ sulfate	6.8 **	3.3 *	ns	
α-Tocopheronic acid sulfate	5.9 *	4.9 *	ns	
CxHyNO Features				
C_4_ H_9_ N O	ns	0.02 ***	48 ***	
C_6_ H_9_ N O	ns	0.05 ***	21 ***	
C_6_ H_11_ N O	ns	0.06 ***	30 ***	
C_6_ H_13_ N O	ns	0.04 ***	44 ***	
C_7_ H_11_ N O	ns	0.01 ***	72 ***	
C_7_ H_13_ N O	ns	0.01 ***	142 ***	

*—*p*-value < 0.05; **—*p*-value < 0.01; ***—*p*-value < 0.001; ns—non-significant.

## Data Availability

The raw data supporting the conclusions of this article will be made available by the authors on request.

## Supplementary Materials

The following supporting information can be downloaded at: https://www.mdpi.com/article/10.3390/metabo14080428/s1, Table S1: Reverse phase chromatographic gradient; Table S2: HILIC chromatographic gradient; Table S3: Putative annotations of significantly changed features; Table S4: The 25 most up/down-regulated features in PA (*p* < 0.001); Table S5: The 25 most up/down-regulated features in MMA (*p* < 0.001); Table S6: The 25 most up/down-regulated features in cblC (*p* < 0.001); Figure S1: **(a**) The levels of feature with MW 74.036, corresponding to propionic acid or it’s isobar; (**b**) the correlation between the feature MW 74.036 and methylmalonic acid in samples; Figure S2: (**a**) Putative structure of propionylcarnitine glycine conjugate; (**b**) Comparison between in-silico predicted MS/MS fragmentations of the putative structure and real MS/MS spectrum. The matching hits are green coloured.

## References

[1] Deodato F., Boenzi S., Santorelli F.M., Dionisi-Vici C. (2006). Methylmalonic and propionic aciduria. Am. J. Med. Genet. Part C Semin. Med. Genet..

[2] Huemer M., Diodato D., Schwahn B., Schiff M., Bandeira A., Benoist J., Burlina A., Cerone R., Couce M.L., Garcia-Cazorla A. (2017). Guidelines for diagnosis and management of the cobalamin-related remethylation disorders cblC, cblD, cblE, cblF, cblG, cblJ and MTHFR deficiency. J. Inherit. Metab. Dis..

[3] Huemer M., Diodato D., Martinelli D., Olivieri G., Blom H., Gleich F., Kölker S., Kožich V., Morris A.A., Seifert B. (2019). Phenotype, treatment practice and outcome in the cobalamin-dependent remethylation disorders and MTHFR deficiency: Data from the E-HOD registry. J. Inherit. Metab. Dis..

[4] Longo N., Sass J.O., Jurecka A., Vockley J. (2022). Biomarkers for drug development in propionic and methylmalonic acidemias. J. Inherit. Metab. Dis..

[5] Maines E., Catesini G., Boenzi S., Mosca A., Candusso M., Strologo L.D., Martinelli D., Maiorana A., Liguori A., Olivieri G. (2020). Plasma methylcitric acid and its correlations with other disease biomarkers: The impact in the follow up of patients with propionic and methylmalonic acidemia. J. Inherit. Metab. Dis..

[6] Forny P., Hörster F., Ballhausen D., Chakrapani A., Chapman K.A., Dionisi-Vici C., Dixon M., Grünert S.C., Grunewald S., Haliloglu G. (2021). Guidelines for the diagnosis and management of methylmalonic acidaemia and propionic acidaemia: First revision. J. Inherit. Metab. Dis..

[7] Manoli I., Gebremariam A., McCoy S., Pass A.R., Gagné J., Hall C., Ferry S., Van Ryzin C., Sloan J.L., Sacchetti E. (2023). Biomarkers to predict disease progression and therapeutic response in isolated methylmalonic acidemia. J. Inherit. Metab. Dis..

[8] Johnson C.H., Ivanisevic J., Siuzdak G. (2016). Metabolomics: Beyond biomarkers and towards mechanisms. Nat. Rev. Mol. Cell Biol..

[9] Wurth R., Turgeon C., Stander Z., Oglesbee D. (2024). An evaluation of untargeted metabolomics methods to characterize inborn errors of metabolism. Mol. Genet. Metab..

[10] Anzmann A.F., Pinto S., Busa V., Carlson J., McRitchie S., Sumner S., Pandey A., Vernon H.J. (2019). Multi-omics studies in cellular models of methylmalonic acidemia and propionic acidemia reveal dysregulation of serine metabolism. Biochim. Biophys. Acta (BBA)-Mol. Basis Dis..

[11] Wikoff W.R., Gangoiti J.A., Barshop B.A., Siuzdak G. (2007). Metabolomics identifies perturbations in human disorders of propionate metabolism. Clin. Chem..

[12] Forny P., Bonilla X., Lamparter D., Shao W., Plessl T., Frei C., Bingisser A., Goetze S., van Drogen A., Harshman K. (2023). Integrated multi-omics reveals anaplerotic rewiring in methylmalonyl-CoA mutase deficiency. Nat. Metab..

[13] Haijes H.A., Jans J.J.M., van der Ham M., van Hasselt P.M., Verhoeven-Duif N.M. (2020). Understanding acute metabolic decompensation in propionic and methylmalonic acidemias: A deep metabolic phenotyping approach. Orphanet J. Rare Dis..

[14] Liu N., Xiao J., Gijavanekar C., Pappan K.L., Glinton K.E., Shayota B.J., Kennedy A.D., Sun Q., Sutton V.R., Elsea S.H. (2021). Comparison of Untargeted Metabolomic Profiling vs Traditional Metabolic Screening to Identify Inborn Errors of Metabolism. JAMA Netw. Open.

[15] Miller M.J., Kennedy A.D., Eckhart A.D., Burrage L.C., Wulff J.E., Miller L.A., Milburn M.V., Ryals J.A., Beaudet A.L., Sun Q. (2015). Untargeted metabolomic analysis for the clinical screening of inborn errors of metabolism. J. Inherit. Metab. Dis..

[16] Warrack B.M., Hnatyshyn S., Ott K.-H., Reily M.D., Sanders M., Zhang H., Drexler D.M. (2009). Normalization strategies for metabonomic analysis of urine samples. J. Chromatogr. B.

[17] Vollmar A.K.R., Rattray N.J.W., Cai Y., Santos-Neto J., Deziel N.C., Jukic A.M.Z., Johnson C.H. (2019). Normalizing Untargeted Periconceptional Urinary Metabolomics Data: A Comparison of Approaches. Metabolites.

[18] Dieterle F., Ross A., Schlotterbeck G., Senn H. (2006). Probabilistic quotient normalization as robust method to account for dilution of complex biological mixtures. application in ^1^H NMR metabonomics. Anal. Chem..

[19] Sun J., Xia Y. (2023). Pretreating and normalizing metabolomics data for statistical analysis. Genes Dis..

[20] Gallagher E.M., Rizzo G.M., Dorsey R., Dhummakupt E.S., Moran T.S., Mach P.M., Jenkins C.C. (2023). Normalization of organ-on-a-Chip samples for mass spectrometry based proteomics and metabolomics via Dansylation-based assay. Toxicol. Vitr..

[21] Barr D.B., Wilder L.C., Caudill S.P., Gonzalez A.J., Needham L.L., Pirkle J.L. (2005). Urinary creatinine concentrations in the U.S. population: Implications for urinary biologic monitoring measurements. Environ. Health Perspect..

[22] Miller R.C., Brindle E., Holman D.J., Shofer J., Klein N.A., Soules M.R., O’connor K.A. (2004). Comparison of specific gravity and creatinine for normalizing urinary reproductive hormone concentrations. Clin. Chem..

[23] Davies S.E., Iles R.A., Stacey T.E., Chalmers R.A. (1990). Creatine metabolism during metabolic perturbations in patients with organic acidurias. Clin. Chim. Acta.

[24] Younessi D., Moseley K., Yano S. (2009). Creatine metabolism in combined methylmalonic aciduria and homocystinuria disease revisited. Ann. Neurol..

[25] Schuck P., Rosa R., Pettenuzzo L., Sitta A., Wannmacher C., Wyse A., Wajner M. (2004). Inhibition of mitochondrial creatine kinase activity from rat cerebral cortex by methylmalonic acid. Neurochem. Int..

[26] Shchelochkov O.A., Manoli I., Sloan J.L., Ferry S., Pass A., Van Ryzin C., Myles J., Schoenfeld M., McGuire P., Rosing D.R. (2019). Chronic kidney disease in propionic acidemia. Anesthesia Analg..

[27] Fowler B., Leonard J.V., Baumgartner M.R. (2008). Causes of and diagnostic approach to methylmalonic acidurias. J. Inherit. Metab. Dis..

[28] Frenkel E.P., Kitchens R.L. (1975). A simplified and rapid quantitative assay for propionic and methylmalonic acids in urine. J. Lab. Clin. Med..

[29] Frenkel E.P., Kitchens R.L. (1977). Applicability of an enzymatic quantitation of methylmalonic, propionic, and acetic acids in normal and megaloblastic states. Blood.

[30] Truscott R.J.W., Pullin C.J., Halpern B., Hammond J., Haan E., Danks D.M. (1979). The identification of 3-keto-2-methylvaleric acid and 3-hydroxy-2-methylvaleric acid in a patient with propionic acidemia. J. Mass Spectrom..

[31] Chalmers R.A., Lawson A.M. (1982). Organic Acids in Man. The Analytical Chemistry, Biochemistry and Diagnosis of the Organic Acidurias.

[32] Wongkittichote P., Cunningham G., Summar M.L., Pumbo E., Forny P., Baumgartner M.R., Chapman K.A. (2019). Tricarboxylic acid cycle enzyme activities in a mouse model of methylmalonic aciduria. Mol. Genet. Metab..

[33] Longo N., Price L.B., Gappmaier E., Cantor N.L., Ernst S.L., Bailey C., Pasquali M. (2017). Anaplerotic therapy in propionic acidemia. Mol. Genet. Metab..

[34] Parfait B., de Lonlay P., von Kleist-Retzow J.C., Cormier-Daire V., Chrétien D., Rötig A., Rabier D., Saudubray J.M., Rustin P., Munnich A. (1999). The neurogenic weakness, ataxia and retinitis pigmentosa (NARP) syndrome mtDNA mutation (T8993G) triggers muscle ATPase deficiency and hypocitrullinaemia. Eur. J. Pediatr..

[35] Subramanian C., Frank M.W., Tangallapally R., Yun M.-K., Edwards A., White S.W., Lee R.E., Rock C.O., Jackowski S. (2021). Pantothenate kinase activation relieves coenzyme A sequestration and improves mitochondrial function in mice with propionic acidemia. Sci. Transl. Med..

[36] Almannai M., El-Hattab A.W. (2021). Nitric Oxide Deficiency in Mitochondrial Disorders: The Utility of Arginine and Citrulline. Front. Mol. Neurosci..

[37] Zecchini V., Paupe V., Herranz-Montoya I., Janssen J., Wortel I.M.N., Morris J.L., Ferguson A., Chowdury S.R., Segarra-Mondejar M., Costa A.S.H. (2023). Fumarate induces vesicular release of mtDNA to drive innate immunity. Nature.

[38] Head P.E., Myung S., Chen Y., Schneller J.L., Wang C., Duncan N., Hoffman P., Chang D., Gebremariam A., Gucek M. (2022). Aberrant methylmalonylation underlies methylmalonic acidemia and is attenuated by an engineered sirtuin. Sci. Transl. Med..

[39] Haijes H.A., van Hasselt P.M., Jans J.J.M., Verhoeven-Duif N.M. (2019). Pathophysiology of propionic and methylmalonic acidemias. Part 2: Treatment strategies. J. Inherit. Metab. Dis..

[40] Roe C., Bohan T. (1982). L-carnitine therapy in propionicacidaemia. Lancet.

[41] Krieger I., Tanaka K. (1976). Therapeutic effects of glycine in isovaleric acidemia. Pediatr. Res..

[42] Rizzo C., Boenzi S., Inglese R., la Marca G., Muraca M., Martinez T.B., Johnson D.W., Zelli E., Dionisi-Vici C. (2014). Measurement of succinyl-carnitine and methylmalonyl-carnitine on dried blood spot by liquid chromatography-tandem mass spectrometry. Clin. Chim. Acta.

[43] Sloan J.L., Achilly N.P., Arnold M.L., Catlett J.L., Blake T., Bishop K., Jones M., Harper U., English M.A., Anderson S. (2020). The vitamin B12 processing enzyme, mmachc, is essential for zebrafish survival, growth and retinal morphology. Hum. Mol. Genet..

[44] Hoss G.R.W., Poloni S., Blom H.J., Schwartz I.V.D. (2019). Three main causes of homocystinuria: CBS, cblC and MTHFR deficiency. What do they have in common?. J. Inborn Errors Metab. Screen..

[45] Sbodio J.I., Snyder S.H., Paul B.D. (2019). Regulators of the transsulfuration pathway. Br. J. Pharmacol..

[46] Stipanuk M.H., Ueki I. (2011). Dealing with methionine/homocysteine sulfur: Cysteine metabolism to taurine and inorganic sulfur. J. Inherit. Metab. Dis..

[47] Mc Guire P.J., Parikh A., Diaz G.A. (2009). Profiling of oxidative stress in patients with inborn errors of metabolism. Mol. Genet. Metab..

[48] Rivera-Barahona A., Alonso-Barroso E., Pérez B., Murphy M.P., Richard E., Desviat L.R. (2017). Treatment with antioxidants ameliorates oxidative damage in a mouse model of propionic acidemia. Mol. Genet. Metab..

[49] Gallego-Villar L., Rivera-Barahona A., Cuevas-Martín C., Guenzel A., Pérez B., Barry M., Murphy M., Logan A., Gonzalez-Quintana A., Martín M. (2016). In vivo evidence of mitochondrial dysfunction and altered redox homeostasis in a genetic mouse model of propionic acidemia: Implications for the pathophysiology of this disorder. Free Radic. Biol. Med..

[50] Liu Y., Wang S., Zhang X., Cai H., Liu J., Fang S., Yu B. (2022). The Regulation and Characterization of Mitochondrial-Derived Methylmalonic Acid in Mitochondrial Dysfunction and Oxidative Stress: From Basic Research to Clinical Practice. Oxidative Med. Cell. Longev..

[51] Richard E., Jorge-Finnigan A., Garcia-Villoria J., Merinero B., Desviat L.R., Gort L., Briones P., Leal F., Pérez-Cerdá C., Ribes A. (2009). Genetic and cellular studies of oxidative stress in methylmalonic aciduria (MMA) cobalamin deficiency type C (*cblC*) with homocystinuria (MMACHC). Hum. Mutat..

[52] Pastore A., Martinelli D., Piemonte F., Tozzi G., Boenzi S., Di Giovamberardino G., Petrillo S., Bertini E., Dionisi-Vici C. (2014). Glutathione metabolism in cobalamin deficiency type C (cblC). J. Inherit. Metab. Dis..

[53] Luo J., Hashimoto Y., Martens L.G., Meulmeester F.L., Ashrafi N., Mook-Kanamori D.O., Rosendaal F.R., Jukema J.W., van Dijk K.W., Mills K. (2022). Associations of metabolomic profiles with circulating vitamin E and urinary vitamin E metabolites in middle-aged individuals. Nutrition.

[54] Wallert M., Schmölz L., Galli F., Birringer M., Lorkowski S. (2014). Regulatory metabolites of vitamin E and their putative relevance for atherogenesis. Redox Biol..

[55] Sharma G., Muller D.P., O’riordan S.M., Bryan S., Dattani M.T., Hindmarsh P.C., Mills K. (2013). Urinary conjugated α-tocopheronolactone—A biomarker of oxidative stress in children with type 1 diabetes. Free Radic. Biol. Med..

[56] Ham Y.-H., Chan K.-K.J., Chan W. (2020). Thioproline Serves as an Efficient Antioxidant Protecting Human Cells from Oxidative Stress and Improves Cell Viability. Chem. Res. Toxicol..

[57] Morellato A.E., Umansky C., Pontel L.B. (2020). The toxic side of one-carbon metabolism and epigenetics. Redox Biol..

[58] Barroso M., Handy D.E., Castro R. (2019). The link between hyperhomocysteinemia and hypomethylation: Implications for cardiovascular disease. J. Inborn Errors Metab. Screen..

[59] Pietzke M., Burgos-Barragan G., Wit N., Tait-Mulder J., Sumpton D., Mackay G.M., Patel K.J., Vazquez A. (2020). Amino acid dependent formaldehyde metabolism in mammals. Commun. Chem..

[60] Tulpule K., Dringen R. (2013). Formaldehyde in brain: An overlooked player in neurodegeneration?. J. Neurochem..

[61] Vaudry H., Ubuka T., Soma K.K., Tsutsui K. (2022). Editorial: Recent Progress and Perspectives in Neurosteroid Research. Front. Endocrinol..

[62] Strac D.S., Konjevod M., Perkovic M.N., Tudor L., Erjavec G.N., Pivac N. (2020). Dehydroepiandrosterone (DHEA) and its Sulphate (DHEAS) in Alzheimer’s Disease. Curr. Alzheimer Res..

[63] Yılmaz C., Karali K., Fodelianaki G., Gravanis A., Chavakis T., Charalampopoulos I., Alexaki V.I. (2019). Neurosteroids as regulators of neuroinflammation. Front. Neuroendocrinol..

[64] Chik M.W., Hazalin N.A.M.N., Singh G.K.S. (2022). Regulation of phase I and phase II neurosteroid enzymes in the hippocampus of an Alzheimer’s disease rat model: A focus on sulphotransferases and UDP-glucuronosyltransferases. Steroids.

[65] Lloyd-Evans E., Waller-Evans H. (2020). Biosynthesis and signalling functions of central and peripheral nervous system neurosteroids in health and disease. Essays Biochem..

[66] Bianchi V.E., Rizzi L., Bresciani E., Omeljaniuk R.J., Torsello A. (2020). Androgen Therapy in Neurodegenerative Diseases. J. Endocr. Soc..

[67] Naylor J.C., Hulette C.M., Steffens D.C., Shampine L.J., Ervin J.F., Payne V.M., Massing M.W., Kilts J.D., Strauss J.L., Calhoun P.S. (2008). Cerebrospinal fluid dehydroepiandrosterone levels are correlated with brain dehydroepiandrosterone levels, elevated in alzheimer’s disease, and related to neuropathological disease stage. J. Clin. Endocrinol. Metab..

[68] Marx C.E., Trost W.T., Shampine L.J., Stevens R.D., Hulette C.M., Steffens D.C., Ervin J.F., Butterfield M.I., Blazer D.G., Massing M.W. (2006). The neurosteroid allopregnanolone is reduced in prefrontal cortex in alzheimer’s disease. Biol. Psychiatry.

[69] Troisi J., Landolfi A., Vitale C., Longo K., Cozzolino A., Squillante M., Savanelli M.C., Barone P., Amboni M. (2019). A metabolomic signature of treated and drug-naïve patients with Parkinson’s disease: A pilot study. Metabolomics.

[70] Shao Y., Li T., Liu Z., Wang X., Xu X., Li S., Xu G., Le W. (2021). Comprehensive metabolic profiling of Parkinson’s disease by liquid chromatography-mass spectrometry. Mol. Neurodegener..

[71] Nuzzi R., Caselgrandi P. (2022). Sex Hormones and Their Effects on Ocular Disorders and Pathophysiology: Current Aspects and Our Experience. Int. J. Mol. Sci..

[72] Nishikawa Y., Morishita S., Horie T., Fukumoto M., Sato T., Kida T., Oku H., Sugasawa J., Ikeda T., Nakamura K. (2017). A comparison of sex steroid concentration levels in the vitreous and serum of patients with vitreoretinal diseases. PLoS ONE.

[73] Schellevis R.L., Altay L., Kalisingh A., Mulders T.W.F., Sitnilska V., Hoyng C.B., Boon C.J.F., Groenewoud J.M.M., de Jong E.K., Hollander A.I.D. (2019). Elevated Steroid Hormone Levels in Active Chronic Central Serous Chorioretinopathy. Investig. Opthalmology Vis. Sci..

[74] Kim J.J., Yu H.G., Ku S.Y. (2010). Sex Steroid Hormone and Ophthalmic Disease. Korean J. Reprod. Med..

[75] Nuzzi R., Scalabrin S., Becco A., Panzica G. (2018). Gonadal Hormones and Retinal Disorders: A Review. Front. Endocrinol..

[76] Onal H., Kutlu E., Aydın B., Ersen A., Topal N., Adal E., Güneş H., Doktur H., Tanıdır C., Pirhan D. (2019). Assessment of retinal thickness as a marker of brain masculinization in children with congenital adrenal hyperplasia: A pilot study. J. Pediatr. Endocrinol. Metab..

[77] Farrell E.K., Chen Y., Barazanji M., Jeffries K.A., Cameroamortegui F., Merkler D.J. (2012). Primary fatty acid amide metabolism: Conversion of fatty acids and an ethanolamine in N18TG2 and SCP cells. J. Lipid Res..

[78] Šebela M., Rašková M. (2023). Polyamine-Derived Aminoaldehydes and Acrolein: Cytotoxicity, Reactivity and Analysis of the Induced Protein Modifications. Molecules.

