# Supplementary material for: Propionic Acidemia, Methylmalonic Acidemia, and Cobalamin C Deficiency: Comparison of Untargeted Metabolomic Profiles
Source: Metabolites. 2024 Aug 2;14(8):428. doi: 10.3390/metabo14080428 (PMC11356709; doi:10.3390/metabo14080428)
Supplement: Supplementary file 1 [file metabolites-14-00428-s001.zip › Figure S1.pptx]

## Slide 1
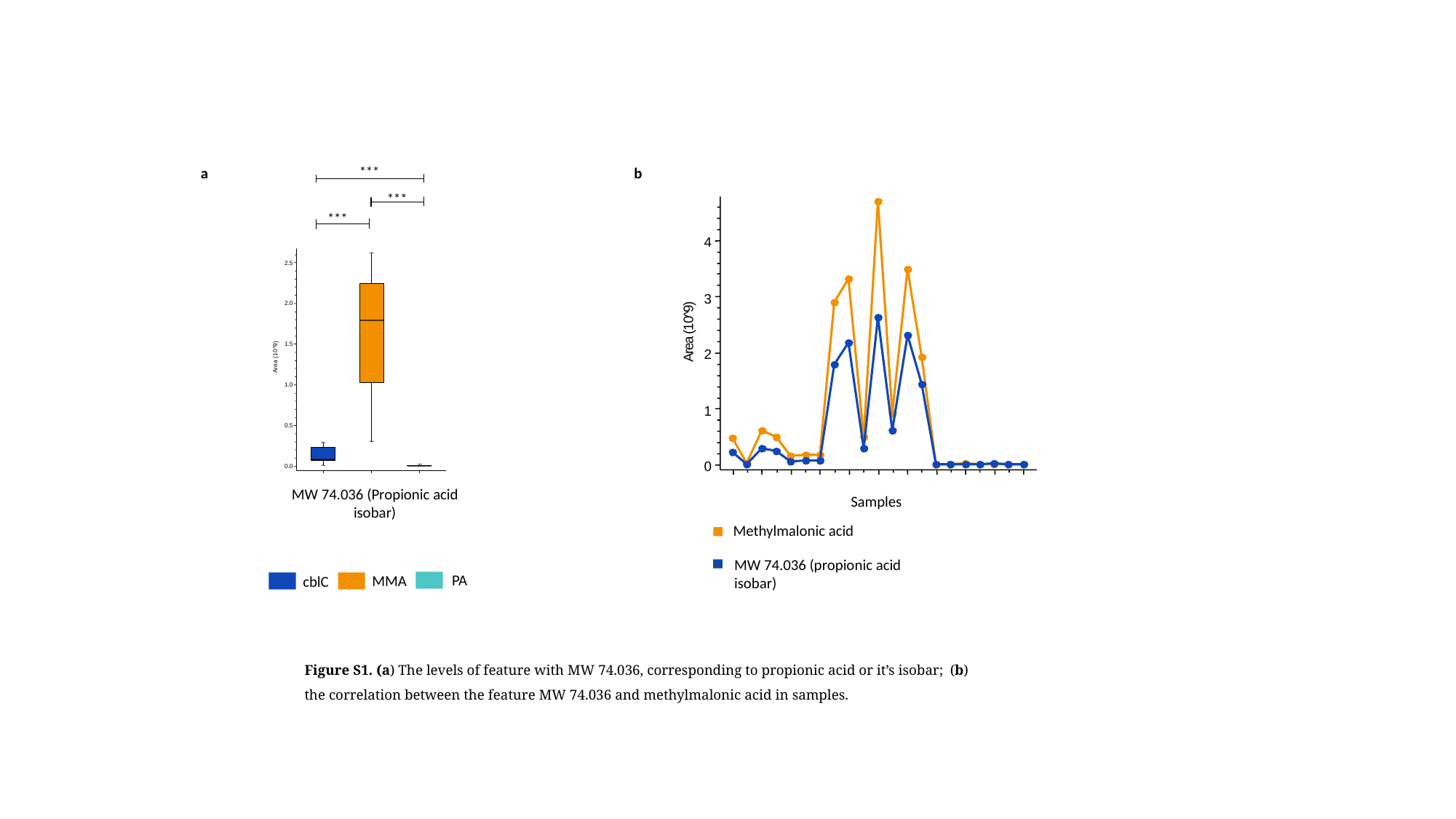

4
3
)
9
^
0
1
(
a
e
r
2
A
1
0
Samples
***
b
a
***
***
2.5
2.0
)
9
1.5
^
0
1
(
a
e
r
A
1.0
0.5
0.0
A
C
A
l
M
b
P
M
C
Groups
MW 74.036 (Propionic acid isobar)
Methylmalonic acid
MW 74.036 (propionic acid isobar)
PA
MMA
cblC
Figure S1. (a) The levels of feature with MW 74.036, corresponding to propionic acid or it’s isobar; (b) the correlation between the feature MW 74.036 and methylmalonic acid in samples.
