# Supplementary material for: Propionic Acidemia, Methylmalonic Acidemia, and Cobalamin C Deficiency: Comparison of Untargeted Metabolomic Profiles
Source: Metabolites. 2024 Aug 2;14(8):428. doi: 10.3390/metabo14080428 (PMC11356709; doi:10.3390/metabo14080428)
Supplement: Supplementary file 1 [file metabolites-14-00428-s001.zip › Figure S2.pptx]

## Slide 1
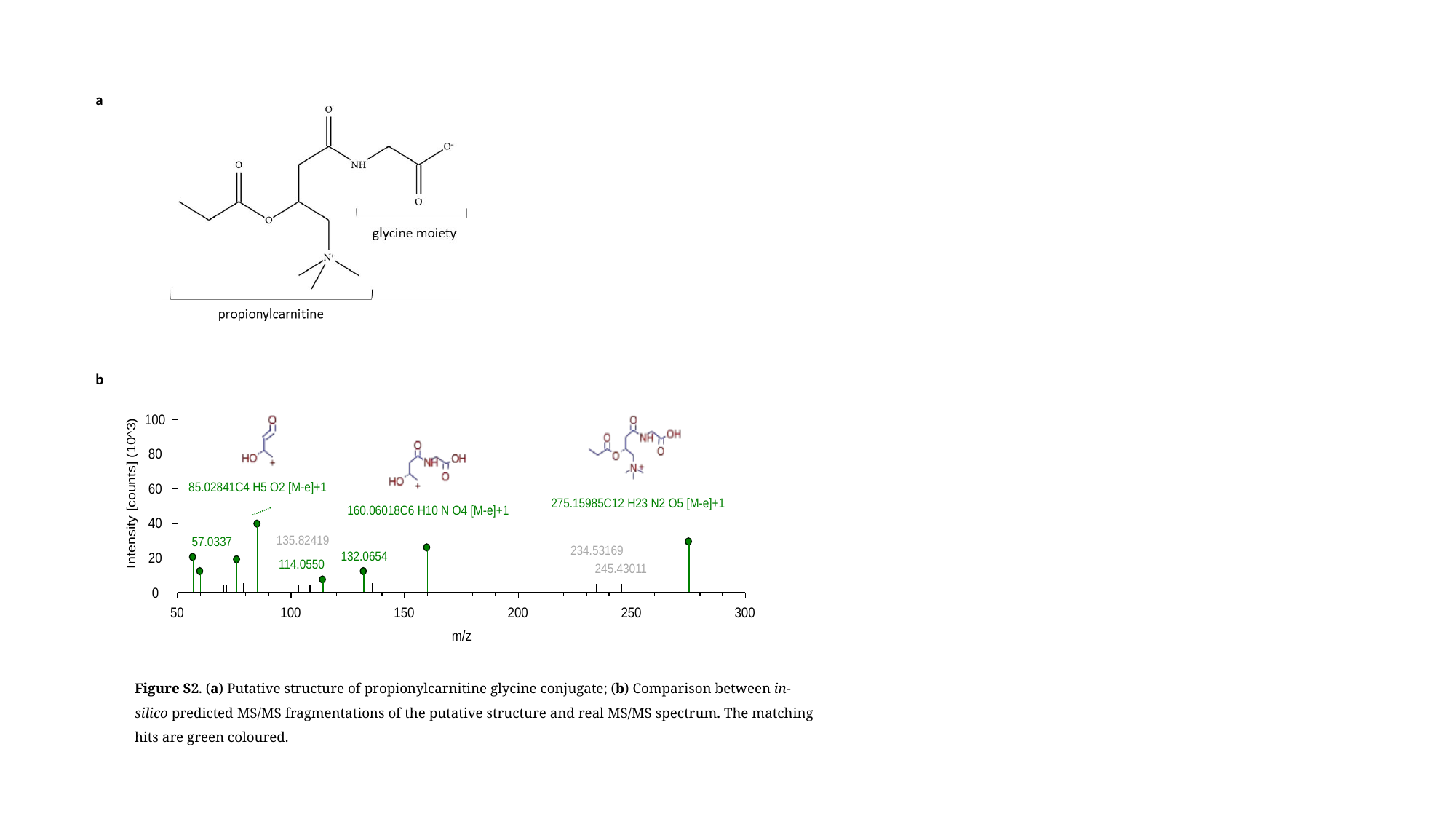

a
b
Figure S2. (a) Putative structure of propionylcarnitine glycine conjugate; (b) Comparison between in-silico predicted MS/MS fragmentations of the putative structure and real MS/MS spectrum. The matching hits are green coloured.
