# Supplementary material for: Propionic Acidemia, Methylmalonic Acidemia, and Cobalamin C Deficiency: Comparison of Untargeted Metabolomic Profiles
Source: Metabolites. 2024 Aug 2;14(8):428. doi: 10.3390/metabo14080428 (PMC11356709; doi:10.3390/metabo14080428)
Supplement: Supplementary file 1 [file metabolites-14-00428-s001.zip › Tables S1-S2.pdf]

Table S1. Reverse phase chromatographic gradient.

Time	Flow ml/min	%B	Curve
0.0	0.3	5.0	5
1.0	0.3	5.0	5
2.0	0.3	20.0	5
12.0	0.3	70.0	5
14.0	0.3	95.0	5
18.0	0.3	95.0	5
18.5	0.3	5.0	5
23.0	0.3	5.0	5

Inject volume 2μL, column temperature 40 ^{0}C.

Table S2. HILIC chromatographic gradient.

Time	Flow ml/min	%B	Curve
0.0	0.5	1.0	5
1.0	0.5	1.0	5
9.0	0.5	95.0	5
10.0	0.5	95.0	5
10.5	0.5	1.0	5
15.0	0.5	1.0	5

Inject volume 2μL, column temperature 30 ^{0}C.
